# Antibiotic production by soil bacteria under aerobic and micro-oxic conditions

**DOI:** 10.17912/micropub.biology.001440

**Published:** 2025-01-31

**Authors:** Cierra Paaaina-Daquioag, Disha Byadarahalli Mohan Kumar, Debra R. Kerr, Leonardo Romero, Kurt M. Regner

**Affiliations:** 1 School of Life Sciences, University of Nevada, Las Vegas, Las Vegas, Nevada, United States

## Abstract

Antimicrobial resistance presents a significant global challenge, undermining the effectiveness of antibiotic therapies and complicating disease management. The origin and spread of antibiotic-resistance genes outpaces the antibiotic discovery process, highlighting an urgent need for new approaches. This study investigated the production of antibiotics by soil bacteria under aerobic and micro-oxic conditions as part of a course-based research experience designed to introduce undergraduates to the global antibiotic resistance crisis. Significant differences in the diameters of the zones of inhibition against three tester strains were observed under differing oxygen concentrations. Soil isolates were identified with 16S rRNA sequence analysis.

**Figure 1. Secondary Antibiosis Screen and Isolate Identification:**
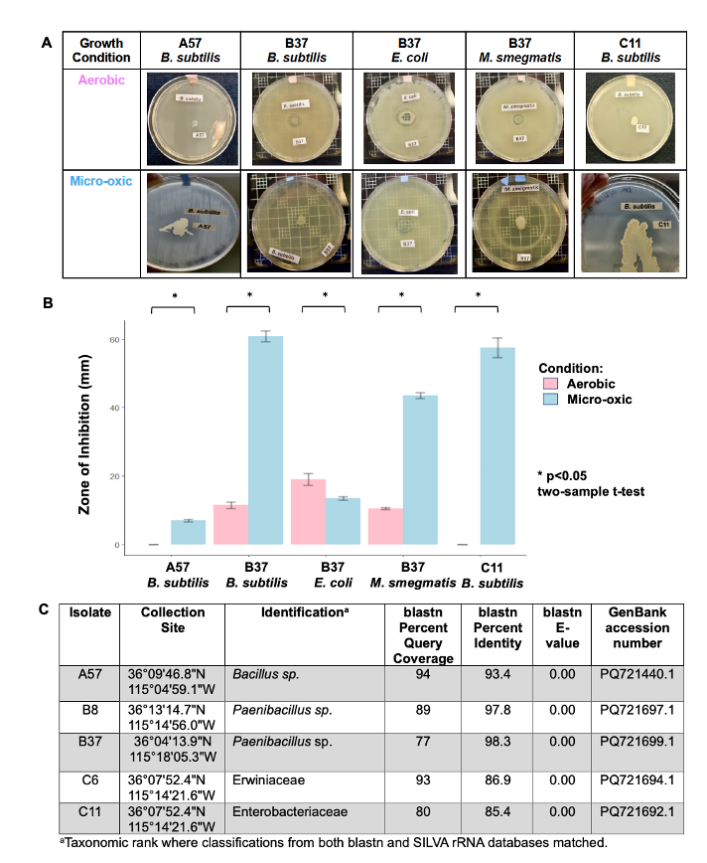
(A) Secondary antibiosis screen: soil isolates were patched over a lawn of a single tester strain ( *B. subtilis, E. coli,* or *M. smegmatis* ), then incubated under aerobic (top) or micro-oxic (bottom) conditions. (B) Statistically significant differences in the inhibition of tester strains by soil isolates when incubated in aerobic (pink) vs micro-oxic (blue) conditions. Values represent the mean of three replicates with error bars representing SEM (two-sample t-test, p<0.05). (C) Identification of the soil isolates using blastn and SILVA rRNA analysis.

## Description

Antimicrobials are prescribed to treat infections, but bacterial and fungal pathogens become resistant due to the misuse of these medications. Consequently, news reports on superbugs such as methicillin-resistant *Staphylococcus aureus* (MRSA), super-gonorrhea, carbapenem-resistant Enterobacteriaceae (CRE) and cephalosporin-resistant *Candida* are all too common. The Centers for Disease Control and Prevention (CDC) estimated that 2.8 million Americans acquire antimicrobial-resistant (ARM) infections, resulting in 35,000 deaths each year (CDC 2019). However, Burham et al. proposed that the number of U.S. deaths due to antimicrobial-resistant infections may be seven times higher due to imprecise medical diagnosis codes (Burnham et al., 2017, 2019) . ARM infections are estimated to increase treatment costs by $4.6 billion in the U.S. (Nelson et al., 2021) . Worldwide, as many as 1.14 million deaths per year were attributed to antimicrobial-resistant bacteria, with a projected increase to 1.91 million by 2050 (Naghavi et al., 2024) .

The discovery of antibiotics in the 20 ^th^ century eliminated infectious diseases as the leading cause of death worldwide. Still, the persistent increase in antimicrobial resistance makes a post-antibiotic world a pending reality (Kwon & Powderly, 2021) . In particular, the ESKAPE pathogens ( *Enterococcus faecium, Staphylococcus aureus, Klebsiella pneumoniae, Acinetobacter baumannii, Pseudomonas aeruginosa and Enterobacter* spp.) are particularly worrisome due to their ability to “escape” being killed by antimicrobials and inciting hospital-acquired infections (Rice, 2008) . Complicating the antimicrobial resistance issue is the expense and painstaking antimicrobial research and development process. In 2017, the estimated cost and time frame between the pre-clinical and post-launch studies was $1.5 billion and 12 years (Towse et al., 2017) .

The microbiology faculty and staff at the School of Life Sciences, University of Nevada, Las Vegas (UNLV) created a course-based research experience to provide undergraduates with a lab experience focused on raising awareness of ARM pathogens. This student-led project investigated antibiotic production under aerobic and micro-oxic conditions. Researching antibiotic production under varying oxygen conditions may facilitate the discovery of novel antimicrobial compounds.

**Soil Collection, Serial Dilution and Bacterial Isolation**

Soil samples collected from gardens, plant beds, and landscaped areas at five private residences (Fig 1 Panel C) were classified as either sandy loam or sandy clay loam (Thien, 1979) and contained between 1.7 × 10⁵ and 6.3 × 10⁸ cfu/g of heterotrophic aerobic bacteria.

**Primary Antibiosis Screen**

A total of 220 soil bacterial strains were randomly selected and screened in antibiosis assays against benign relatives of ESKAPE pathogens (listed in the Reagents section). Of these, five isolates inhibited at least one of the tester strains under micro-oxic conditions and were selected for the Secondary Antibiosis Screen.

**Secondary Antibiosis Screen**

Five soil isolates were selected for antibiosis assays with *Mycobacterium smegmatis* , *Escherichia coli* , and *Bacillus subtilis* under aerobic and micro-oxic conditions. Three isolates (A57, B37, C11) produced significantly larger zones of inhibition under micro-oxic conditions. Isolate B37 produced significantly larger zones against *B* . *subtilis* and *M* . *smegmatis* under micro-oxic conditions and a larger zone against *E* . *coli* under aerobic conditions. Isolates A57 and C11 did not inhibit *B* . *subtilis* under aerobic but did so in micro-oxic conditions. Isolate B8 inhibited *M* . *smegmatis* under both aerobic and micro-oxic conditions but there was no significant difference. There was also no significant difference in the inhibition of the tester strains under aerobic versus micro-oxic conditions for isolate C6. Representative images and the mean zones of inhibition (mm) ± standard error appear in Fig 1 Panel A-B.

**Colony PCR, Gel Electrophoresis and Isolate Identification**

A DNA band of approximately 1,000 bp confirmed a successful PCR amplification of the 16S rRNA gene for all soil isolates. The identities of the soil isolates based on blastn and SILVA rRNA analysis of the partial 16S rRNA sequences appear in Fig 1 Panel C.

The soil samples contained large populations (cfu/g) of heterotrophic bacteria capable of growing on 10% TSA at room temperature. We chose these conditions to promote the isolation of a diverse bacterial population by slowing growth by reducing nutrients and using a cooler temperature. The soil isolates that grew in the anaerobic culture jars with a GasPak (Becton Dickinson, Co.) are assumed to be either facultative anaerobes or microaerophiles. A limitation of this research was our inability to measure the atmospheric O _2_ concentration within the anaerobic jar. The growth of *M* . *smegmatis* , which can function as a microaerophile, indicates that a small percentage of oxygen remained in the anaerobic jars. For these reasons, the authors used the term micro-oxic (<21% O _2_ ) rather than anaerobic. In all experiments, the methylene blue oxygen indicator strips remained white.

The Secondary Antibiosis Screen revealed differential antibiotic production under micro-oxic conditions, as evidenced by significantly larger zones of inhibition against the tester strains. It is well documented that O _2_ concentration influences bacterial gene expression (Iuchi & Lin, 1993) , but the increased zones of inhibition under micro-oxic conditions may be attributed or partially attributed to slower growth rates of the tester strains rather than enhanced antibiotic production. Each tester strain possesses at least one mechanism for surviving in low-oxygen environments: *B. subtilis* can utilize nitrate respiration or fermentation, *E. coli* functions as a facultative anaerobe, and *M. smegmatis* is capable of microaerophilic growth (Logan & Vos, 2015; Scheutz & Strockbine, 2015; Magee & Ward, 2015) . Additional research is needed to determine if reduced growth rates under micro-oxic conditions may have influenced the size of the inhibition zones, as this relationship could significantly impact the interpretation of the antibiotic production results.

The identities of the five soil isolates, determined through partial 16S rRNA gene sequence analysis, are consistent with bacterial genera known to produce antimicrobial compounds. Bacilli, including members of the genus *Paenibacillus* , are well-documented to produce a wide range of antimicrobial compounds (Grady et al., 2016; Mannanov & Sattarova, 2001) . Two isolates, C6 and C11, showed conflicting taxonomic classifications between blastn and SILVA analyses. For C6, blastn identified it as *Pantoea* sp., while SILVA placed the isolate in the Erwiniaceae. Similarly, C11 was classified differently by each platform - blastn identified it as *Klebsiella* while SILVA named it *Enterobacter* . Due to the inconsistency, C11 was identified as a member of the Enterobacteriaceae. Members of the family Enterobacteriaceae, which includes the Erwiniaceae, are documented to produce numerous antimicrobial compounds (Mohite et. al, 2022) . Future research will focus on isolating the antimicrobial compounds and examining the genomes of the soil isolates for antibiotic biosynthetic pathways.

## Methods

**Soil Collection, Serial Dilution and Bacterial Isolation**

Soil was collected from private residences in Clark, Co., NV (Fig 1 Panel C) and the *texture-by-feel method* was used to describe the soil type (Thien, 1979) . The GPS coordinates of the collection sites were determined with Google Maps ( *Map of the Las Vegas Metropolitan Area, Clark County, Nevada* , 2024). A 10-fold dilution series was performed with sterile deionized water, and dilutions 10 ^-3^ through 10 ^-7^ were plated on 10% Tryptic Soy Agar (BD Difco, Franklin Lakes, NJ) with cycloheximide (25 μg/ml). Two sets of plates were prepared, with one incubated in EZ Container anaerobic culture jars with a GasPak and dry methylene blue indicator strip (Becton Dickinson, Co.). All plates were incubated at room temperature, and colonies were randomly selected on day 7 for aerobic and day 11 for micro-oxic treatments.

**Primary Antibiosis Screen and Streak Plate Protocol**

A turbid culture of benign relatives of the ESKAPE pathogens ( *Escherichia coli, Staphylococcus epidermidis, Klebsiella aerogenes, Pseudomonas aeruginosa* , *Bacillus subtili* s, *Pectobacterium carotovorum* ) was spread on the surface of 10% TSA plates with a sterile cotton swab. Soil isolates from the serial dilution plates were transferred using a sterile loop in a grid pattern onto each ESKAPE plate. Two sets of plates were produced, with one incubated aerobically and the other under micro-oxic conditions. Soil isolates that produced a zone of inhibition against any ESKAPE relative tester strain were streaked for single colonies on full-strength TSA plates three successive times. For the second and third dilution streaks, a single well-isolated colony was selected. As stated above, two sets of plates were generated, with one incubated aerobically and the other under micro-oxic conditions at room temperature.

**Micro-oxic Medium Preparation**

All TSA plates used for evaluating micro-oxic growth in the Secondary Antibiosis Screen (described below) were prepared as follows. As per the manufacturer's instructions, the TSA was mixed with deionized water, sterilized (45 min, 121℃ 20 psi), and after cooled in a water bath (55℃), poured into 100 x 15 mm polystyrene Petri dishes. Immediately after pouring, the plates were placed in clean, EZ Container anaerobic culture jars and allowed to solidify at room temperature, checked for contamination, inverted, and stored with the addition of a GasPak and indicator strip.

**Secondary Antibiosis Screen**

Soil isolates that produced zones of inhibition against any of the seven tester strains in the Primary Antibiosis Screen in micro-oxic conditions were selected for further evaluation. In the Secondary Antibiosis Screen, a turbid broth culture of *Bacillus subtilis* (Gram-positive), *Escherichia coli* (Gram-negative), and *Mycobacterium smegmatis* (Acid-fast) was spread on 10% TSA plates using sterile cotton swabs. A single colony of each soil isolate was spotted in the center of the plate using a sterile inoculation loop. Two sets of plates were prepared for each isolate: one for aerobic incubation and another set for micro-oxic incubation. Three replicates were made for each of the tester strains. The diameter of the zone of inhibition (mm) was measured using a ruler. The zones of inhibition for the aerobic plates were measured after 3 days of incubation at room temperature. The incubation time for the micro-oxic treatments varied from 5 to 9 days to allow sufficient time for a visible bacterial lawn of the tester strains ( *B. subtilis* , *M. smegmatis* , *E. coli* ).

**Colony PCR and Gel Electrophoresis**

A single well-isolated bacterial colony was transferred to 100 µL of sterile water, vortexed, and incubated at 95°C for 5 min. The bacterial suspension was vortexed again, and 6 µL was added to 40µL of OneTaq Hot Start Quick-Load 2x Master Mix (New England Biolabs, Ipswich, MA) containing the forward primer and reverse primers. The bacterial 16S rRNA gene was amplified using the universal forward 357f (5’CTCCTACGGGAGGCAGCAG-3’) and reverse 1391r (5’-GACGGGCGGTGTGTRCA-3’) primers (ThermoFisher Scientific, Waltham, MA). The PCR conditions were as follows: a cell lysis and Hot Start Taq activation for 10 min at 94°C (105°C lid temp), followed by 25 cycles of denaturation for 30 sec at 94°C, annealing for 30 sec at 58°C and elongation for 80 sec at 72°C for 25 cycles. A final extension step of 10 minutes at 72°C was included to complete the amplification.

The PCR product (10 μL) was loaded into a 1% agarose gel with Midori green advance (Bulldog-Bio, Portsmouth, NH) and run at 48 V for 45 minutes using the blueGel™ electrophoresis system (miniPCRbio, Cambridge, MA) containing 30 mL of 1X TBE buffer. The outer wells of the gels were not loaded, and the second from the left was reserved for 10 μL of a 1.0 kb Quick Load Purple DNA ladder (New England Biolabs, Ipswich, MA).

**DNA Sequencing, AB1 Conversion and Sequence Analysis**

PCR product clean-up and Sanger sequencing were performed by Functional Biosciences (Madison, WI). DNA chromatograms were generated with Poly Peak Parser and the Bugaco Sequence Conversion Tool was employed for AB1 conversion to FASTA files ( *Bugaco Online Sequence Conversion Tool* , 2020; Hill et al., 2014). Soil isolate identification was performed using the nucleotide Basic Local Alignment Search Tool (blastn) and the SILVA rRNA database (v. 138.2) (Altschul et al., 1990; Quast et al., 2012) . To ensure high-confidence results, only blastn alignments with a minimum of 95% sequence identity and greater than 40% query coverage were considered. In cases where different species of the same genus had identical values for these criteria, we reported only the genus. We compared taxonomic classifications from blastn and the SILVA least common ancestor output. For each sequence, we report the taxonomic rank that both systems agreed upon. Default values were used for all programs. The GenBank accession numbers are reported.

**Statistical Analysis**

Two sample t-tests were performed with R 4.2.2 (R Core Team, 2021) . Statistical significance indicates p < 0.05.

## Reagents

Glycerol stocks of the bacterial strains used in this study are stored in the microbiology teaching lab and -80°C freezer room, Juanita Greer White Hall, School of Life Sciences, UNLV.

**Table:** 

**Primary Screen Tester Strains**	**Source**
*Escherichia coli* ATCC 25922	Tiny Earth Initiative ^a^
*Staphylococcus epidermidis* ATCC 14990	Tiny Earth Initiative
*Klebsiella aerogenes* ATCC 25922	Tiny Earth Initiative
*Pseudomonas putida*	Tiny Earth Initiative
*Bacillus subtilis* ATCC 6051	Tiny Earth Initiative
*Pectobacterium carotovorum* ATCC 25270	Tiny Earth Initiative
	
**Secondary Screen Tester Strains**	
*Bacillus subtilis* ATCC 6051	Tiny Earth Initiative
*Escherichia coli* ATCC 25922	Tiny Earth Initiative
*Mycobacterium smegmatis* ATCC 700084	Tiny Earth Initiative

^a^ Tiny Earth Initiative, University of Wisconsin-Madison
